# Homonuclear nickel(II) metal-organic framework-type supramolecule: adsorption and emission properties

**DOI:** 10.55730/1300-0527.3452

**Published:** 2022-05-12

**Authors:** Idongesit Justina MBONU, Olusegun Khinde ABIOLA, Hamzah Audu BAWA

**Affiliations:** Department of Chemistry, Federal University of Petroleum Resources, Effurun, Nigeria

**Keywords:** Adsorption, emission properties, nickel (II) MOF-type supramolecule

## Abstract

Coordination-driven self-assembly of nickel (II) ions with equimolar amounts of 1, 10 phenanthroline, and benzoic acid was used to create homonuclear nickel (II) MOF-type supramolecule. Single crystal X-ray diffraction, scanning electron microscopy, differential scanning calorimetry, photoluminescence measurement, FT-IR, UV-visible spectroscopy, and nitrogen physisorption measurement were used to characterize the compound. The single crystal X-ray diffraction study reveals that the synthesized compound is two-dimensional with pores in the structure. The homonuclear nickel (II) MOF-type supramolecule crystallized in a triclinic crystal system, with space group P-1 (No.2) and unit cell parameters a = 9.2053(4) Ǻ, b = 13.2964(5) Ǻ, c = 15.8998(6) Ǻ, α = 66.296(2) °, β = 89.400(2) °, γ = 89.441(2) °. The crystal structure result shows π-π and intermolecular interactions between adjacent 1, 10-phenanthroline molecules. DSC result showed a gradual decomposition confirming thermal stability of the compound. The emission (468 nm) spectrum result shows energy peaks of a typical multiphonon phase, indicating luminescence process. The surface topology and single absorption maximum at 308 nm reveal formation of a distorted octahedral structure. Brunauer–Emmett–Teller and Langmuir surface area results 383.741 and 975.830 m^2^g^−1^, respectively, indicate high adsorption capacity. Langmuir isotherm model result of the compound shows an efficient adsorption property for storing energy. The excellent nickel (II) MOF-type supramolecule adsorption capacity and emission property opened the door to its use in energy storage applications especially in photovoltaic cells.

## 1. Introduction

Adsorption and emission are important in today’s society and industry. Adsorption technologies are widely used in processes ranging from water and environmental treatment to nuclear fuel enrichment and electronics. Emission techniques are used in industries such as solar cells and pharmaceutical production. Porous supramolecular materials formed through covalent bonding in coordination-driven self-assembly [1–8] have received a lot of attention in this frame of adsorption and emission because of their large surface area, artificially controlled pore size distribution, high thermal and chemical stability, and inexhaustible functional groups. Because of these properties, supramolecules are effective adsorbents for variety of compounds. As stand-in sorbents [9], these molecular compounds make excellent test materials for pretreatment strategies in industries. Dynamic responses to oil spills in open waters have resulted in the use of sorbent materials such as dispersants, skimming, herders, and in situ burning due to environmental concerns and threats to human health. Natural sorbents lose separation selectivity and performance due to biodegradation and absorption of both oil and water; thus, their properties change with storage prior to use. Many attempts have been made to replace them with synthetic sorbents, which are an efficient, cost-effective, and environmentally friendly method of controlling oil spills in terms of cleanups and posttreatment of oil-loaded sorbents [1–3].

Stang and colleagues [10] developed a comprehensive synthetic method for self-assembly that has been utilized successfully [5] to build a range of discrete porous 2D or 3D supramolecules that can be used as sorbents. To self-assemble, a ligand with donor properties and metal-containing acceptors are fixed at appropriate bond angles, resulting indiscrete supramolecular architectures with a high quantitative yield. The success of this method can be attributed to the thermodynamic regulation of coordination between the metal-acceptor and ligand donor; this condition favors the enthalpy formation of discrete entities with specified sizes, shapes, and functional groups [7]. We used metal-ligand ‘coordination-driven self-assembly’ strategies in conjunction with weak interactions such as π-π stacking, electrostatic, and van der Waals forces to create 2D nickel supramolecular of defined size and shape. As shown in Scheme 1, we chose to test the homonuclear nickel (II) MOF-type supramolecule on various adsorption models for specific adsorption performance and photoluminescence properties for energy storage applications.

## 2. Materials and methods

### 2.1. Materials

All the chemicals used in this work (benzoic acid, 1, 10-phenanthroline, K_2_CO_3_, and NiSO_4_.6H_2_O) were purchased from Sigma-Aldrich. These chemicals were of high purity, and they were used as obtained. To determine the elemental composition of homonuclear nickel (II) MOF-type supramolecule, PerkinElmer 2400 [11–13] elemental analyzer was used. The morphology of the synthesized compound was investigated by scanning electron microscopy [14] using a Zeiss Supra [B] 55 instruments equipped with a Gemini column and high-efficiency secondary electron detector operating at 15 kV/5 nm [11]. Nicolet Magna **[D]-** FT-IR spectrometer ((4000–650 cm^−1^) in attenuated total reflection mode with SMART ORBIT accessory was used to determine the functional groups absorption bands within homonuclear nickel (II) MOF-type supramolecule. This was done to confirm coordination between the metal-acceptor and ligand donors. The hydrated nickel (II) MOF-type supramolecule compound was subjected to differential scanning calorimetry (DSC) analysis using PerkinElmer DSC Instrument in an inert nitrogen gas flow environment between 25 and 400 °C in order to ascertain the thermal stability, decomposition of this new compound under heating. Single crystal structure of the nickel (II) MOF-type supramolecule compound was examined utilizing Bruker DUO APEX II [E] CCD diffractometer using graphite-monochromatic Mo *kα.*(λ = 0.71071 Ǻ) with an Oxford Cryostream-700. A UV-visible spectrophotometer was used to determine the differing energy levels of the elements within nickel (II) MOF-type supramolecule.

### 2.2. Method of synthesis of homonuclear nickel (II) MOF-type supramolecule

To a 50 mL flask equipped with a reflux condenser was added benzoic acid (1.39 mmol), 1, 10-phenanthroline (0.50 mmol), K_2_CO_3_ (13.91 mmol), and NiSO_4_.6H_2_O (0.069 mmol) in degassed methanol (25 mL). The mixture was stirred at 60 °C for about 4 h[13]. After cooling to room temperature, the solution formed was evaporated for crystallization. Blue crystals were obtained after 3 days; yield: 64%. Elemental analyses show that the C, H, Ni, and O are 16.30, 5.90, 12.7, 13.30, 44.50, 7.30 (calc.) and 15.98, 5.60, 12.76, 13.0, 45.60, and 7.06 (found), respectively.

### 2.3. Adsorption analysis

Quntachrome Instrument was used to investigate the physisorption measurement using nitrogen gas at 77 K. Nitrogen sorption isotherms were measured at 77 K on the nickel (II) MOF-type supramolecule [10] to evaluate the pore size **[**9], specific surface area [12], pore volume, and pore size distribution [6]. To get rid of guest solvent molecules, the compound was degassed at room temperature for 24 h before obtaining sorption isotherms measurements. The surface area [12] was evaluated utilizing Brunauer–Emmett and Langmuir models of nitrogen adsorption/desorption measurements at liquid nitrogen [12] temperature of −196 °C [7].

## 3. Results and discussion

**Figure 1:**
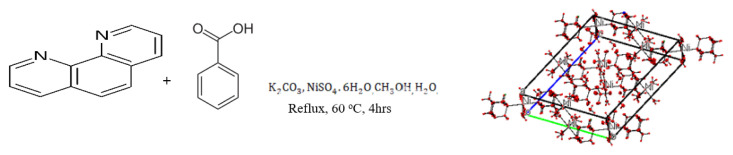
Graphical reaction pathway on how the nickel (II) MOF-type supramolecule was synthesized.

Figure 1 gives a graphical reaction pathway on how the homonuclear nickel (II) MOF-type supramolecule was synthesized.

**Figure 2:**
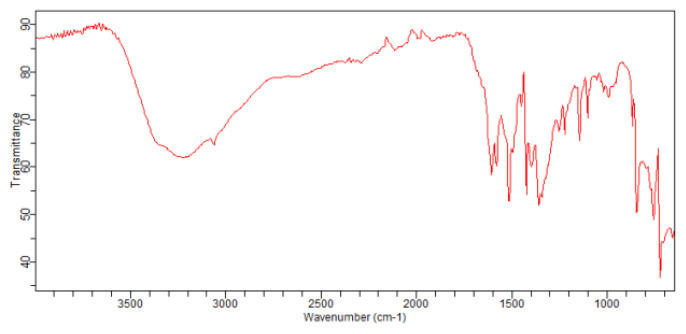
FTIR spectrum of homonuclear nickel (II) MOF-type supramolecule.

Figure 2 shows FTIR spectrum of nickel (II) supramolecule. The absorption bands observed within the range of 3700–3100 and 2133 cm^−1^ [1, 13] are the stretching and bending peaks of O–H of H_2_O molecule, respectively. The peak at 1750 cm^−1^ correspond to C=O bond. A good deal of absorption vibration peaks appears within the range of 1000–500 cm^−1^ region of the nickel (II) supramolecule crystals.

**Figure 3:**
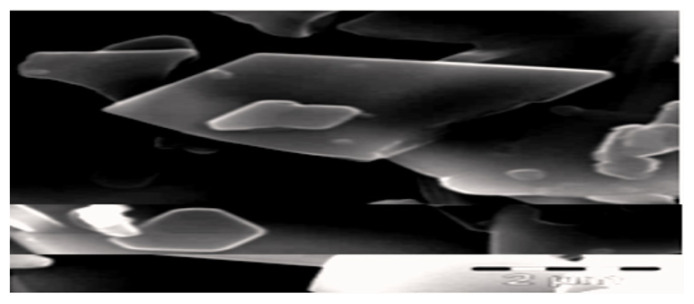
SEM image of homonuclear nickel (II) MOF-type supramolecule.

Figure 3 is a SEM micrograph of the homonuclear nickel (II) MOF-type showing variations in morphology [15]. The SEM micrograph shows the particles are of irregular shapes with smaller truncated rectangular structures. However, a few 2 μm enlarged particles were seen within the micrograph of compound, which may be caused by the amassing of smaller particles. The irregular-like structures are shaped as a result of even self-assembly of the truncated rectangles and after achieving a threshold measure of the rectangular-like structure, the growth stops and the unused develops into truncated features.

**Figure 4:**
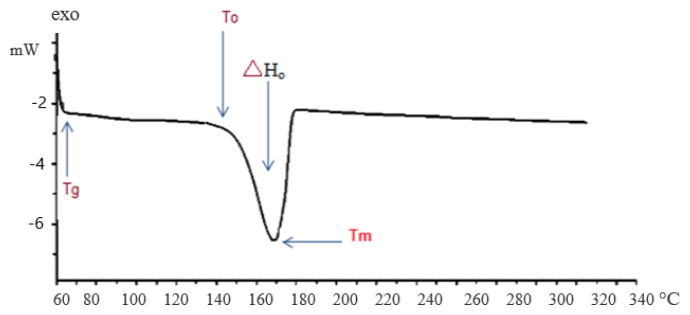
Thermogram of homonuclear nickel (II) MOF-type supramolecule The peak melting temperature of 170 °C, enthalpy of fusion =ΔH_o_, T_o_= onset of temperature of melting, T_m_ = Peak of temperature of melting.

Figure 4 is the DSC thermogram that occurred within the compound in a sigmoidal baseline curve of endothermic transition. The differential scanning calorimetric heat flux thermal analysis is utilized to investigate polymorphism, storage conditions, and shelf life of the compound. The compound melted at 170 °C, the sharpness in melting is an indication of the degree of purity of this compound. The glass transition [13] state observed at ~65 °C shows a step-wise increase in the heat capacity (ΔH = 823.46 J/g, 159.92 KJ/mol) of this compound. The compound is stable before and after 170 °C.

**Figure 5:**
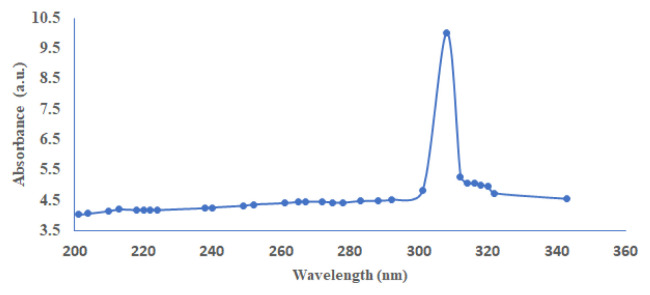
Absorption spectrum of nickel (II) MOF- type supramolecule.

**Figure 6:**
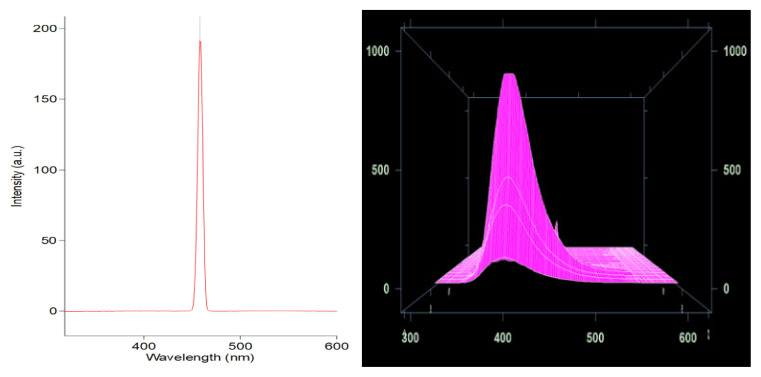
a) Emission and b) normalized emission spectra of nickel (II) MOF-type supramolecule.

The Uv-visible spectrum in Figure 5 shows just a single maximum (308 nm) assigned to absorption band of Ni^2+^ octahedral complexes in ^3^A_2g_ → ^3^T_1g_(^3^P) energy level [14]. Figures 6a and 6b are emission spectra of the synthesized compound at 468 nm.

The electronic transition associated with 308 nm (Figure 5) absorption and 468 nm (Figures 6a and 6b) emission show there is a large stroke shift, implying a rapid relaxation from the absorption to the emissive state. This intermolecular energy transfer shows that part of the molecule acts as a donor, absorbing light, and another portion of the molecule acts as an acceptor, which emits light with significant red shifts [15 – 16]. The Ni^2+^ centers have vacant d-orbitals making them good electron acceptors. When reacted with electron donors such as 1,10-phenanthroline and benzoic acid, these molecules form electronic dipoles that facilitate donor-acceptor charge transfer upon excitation with light. As a result, donor-acceptor nickel(II) displayed a large stoke shift which is an indication of an enhanced luminescence property.

**Figure 7:**
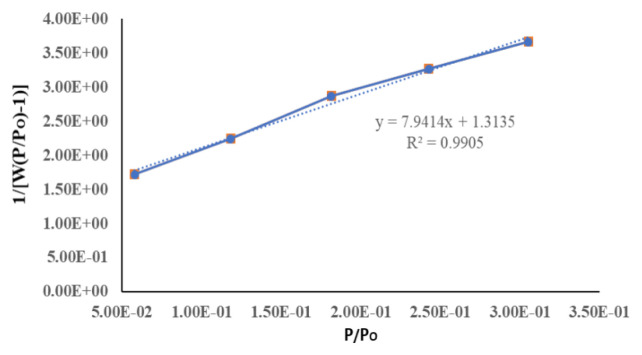
BET nitrogen adsorption isotherm of nickel (II) supramolecule.

**Figure 8:**
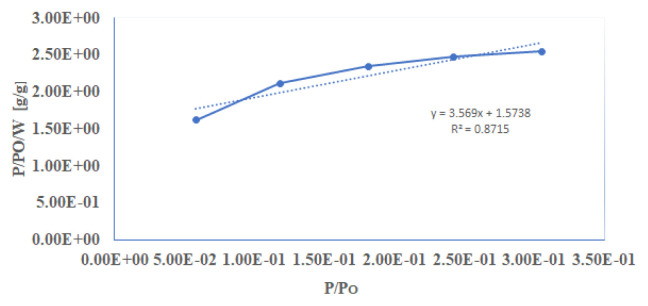
Langmuir plot of N_2_ adsorption at 77.3K

**Figure 9:**
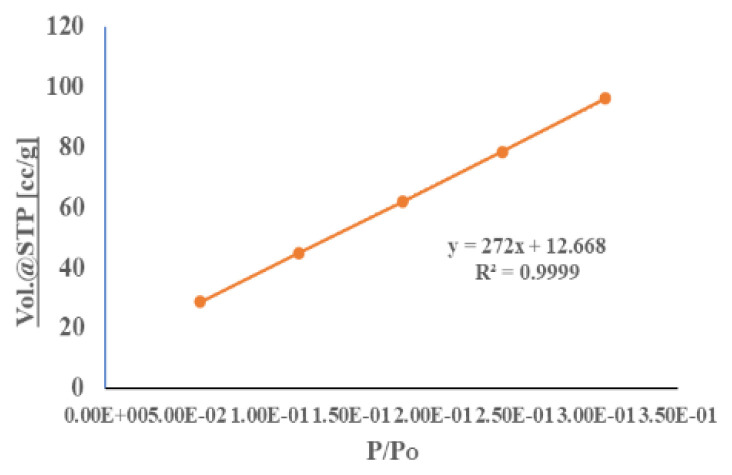
A linear form of Langmuir plot of compound.

Figures 7–9 depict N_2_ adsorption/desorption isotherms at 77 K by Brunauer–Emmett–Teller (BET) and Langmuir, respectively (Table 1). The surface area analyses from N_2_ adsorption isotherm by BET and Langmuir were 383.741 and 975.830 m^2^g^−1^, respectively. The Dubinin–Radushkevch model yielded a median pore diameter of 5.571 Å, which was consistent with that obtained from single crystal structure analysis. The correlation coefficients (R^2^ = 0.991) and surface area using the BET and Langmuir model were determined by the following expression in Eq. 1 [1] **:**


(1) $$\frac{1}{W\left[\left(\frac{P_{o}}{P}-1\right)\right]}=\frac{1}{WmC}+\frac{C-1}{W_{m}C}\left(\frac{P_{o}}{P}\right).$$

Here W = weight of gas adsorbed, P/P_0_ = relative pressure, W_m_ = weight of adsorbate as a monolayer, C = constant. Slope (s), intercept (i), and weight (Wm) were determined from Eq. 2 [1] below:


(2) $$s=\frac{C-1}{W_{m}C}i=\frac{1}{W_{m}C}W_{m}=\frac{1}{s+i}.$$

Total surface area (St) was calculated from expression (Eq.3):


(3) $$S_{t}=\frac{W_{m}NA_{CS}}{M}.$$

**Figure 10:**
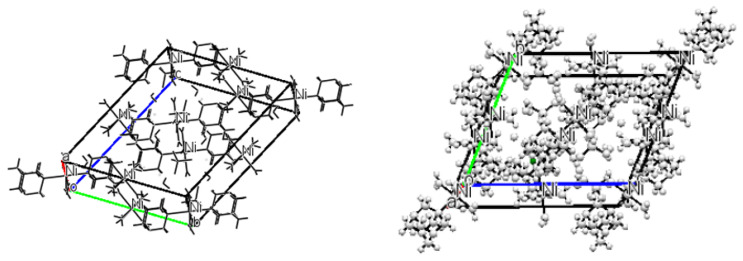
Packing along default view ‘b’.

The single X-ray study was used to explore the crystal structure of nickel (II) supramolecule compound and the results are shown in Figures 10a and 10b with crystal packing along default view of “b” axis. The compound C_12_ H_52_ N_8_ Ni_2_ O_24.55_ S_2_ crystallizes in a triclinic crystal system, with space group P-1(No.2) and unit cell parameters a = 9.2053(4) Ǻ, b= 13.2964(5) Ǻ, c = 15.8998(6) Ǻ, α = 66.296(2) °, β = 89.400(2) °, γ = 89.441(2) °. Detailed crystal data, structure refinement, and conditions for data collection are given in Table 2. The geometry of the compound was evaluated utilizing the program PLATON [1]. Mercury software was used to generate the molecular and packing diagrams. Figure 10 is the ORTEP plot of the compound ellipsoids [7] drawn at 50% probability. The torsion angle, bond angles, and bond distances are as given in Tables S1 and S2, respectively. Nickel complex is octahedrally coordinated having two bidentate carboxylates and bis-coordinating 1, 10-phenanthroline. The Ni-O bond distances are in the range of 2.0322(9) to 2.0239(11) Ǻ and the two Ni-N are 2.3004(12) and 2.2920(12) **Ǻ**, respectively, showing their bis-coordination modes to the nickel ion [1, 14, 16].

## 4. Conclusion

The coordination-driven self-assembled method was used to synthesize porous photoluminescence-adsorbent compound with a high surface area of 975.830 m^2^/g and a large pore volume of 0.64 cm^3^g^−1^. We characterize this compound by DSC, SEM, single crystal X-ray diffraction studies, FTIR, UV-visible and photoluminescence spectroscopy. This compound displayed a high sorption capacity under some adsorption isotherms and high energy storing capacity through experimental findings. The compound displayed a large stroke shift. These findings were consistent with a number of authors’ literature reviews and adsorption experiments. These experimental results demonstrated that homonuclear nickel (II) MOF-type supramolecule has high adsorption capacity, high surface area, and good thermal stability for storing energy and can also be used in deep denitrogenation of liquid hydrocarbons streams from refinery streams via adsorption.

**Table S1.** Final coordinates and equivalent isotropic displacement parameters of the nonhydrogen atoms.

Atom	x	y	z U(eq)	[Ang^2]
Ni1	0	1/2	1/2	0.0127(1)
Ni2	1/2	0	0	0.0132(1)
Ni3	1/2	0	1/2	0.0153(1)
Ni4	0	1/2	0	0.0152(1)
S5	0.02616(4)	0.86428(3)	0.17937(2)	0.0199(1)
O11	−0.21623(10)	0.47126(9)	0.52313(7)	0.0207(3)
O12	0.05064(11)	0.37945(8)	0.62294(7)	0.0206(3)
N11	−0.00088(12)	0.37223(9)	0.43638(7)	0.0142(3)
N12	−0.09975(13)	0.19488(10)	0.44908(8)	0.0197(3)
N13	0.15019(13)	0.24234(10)	0.39790(8)	0.0198(3)
N14	−0.05015(13)	0.33878(10)	0.29786(8)	0.0193(3)
S6	0.53041(4)	0.63260(3)	0.32017(2)	0.0193(1)
C11	−0.09871(15)	0.27827(11)	0.48825(9)	0.0185(4)
C12	0.14770(15)	0.32593(12)	0.43709(10)	0.0187(4)
C13	−0.05078(15)	0.42018(11)	0.33904(9)	0.0176(3)
C14	0.04928(16)	0.15345(12)	0.45056(10)	0.0218(4)
C15	0.09825(16)	0.29425(12)	0.30259(10)	0.0214(4)
C16	−0.14703(16)	0.24804(12)	0.35247(10)	0.0213(4)
O21	0.28589(11)	0.03144(10)	−0.03072(8)	0.0253(3)
O22	0.56719(13)	0.11101(9)	−0.12389(7)	0.0256(3)
N21	0.50378(12)	0.12807(9)	0.06254(7)	0.0146(3)
N22	0.35799(13)	0.25653(10)	0.10597(9)	0.0207(3)
N23	0.60573(13)	0.30441(10)	0.04974(8)	0.0205(3)
N24	0.56276(13)	0.15911(10)	0.20113(8)	0.0197(3)
C21	0.35610(15)	0.17430(12)	0.06571(10)	0.0190(4)
C22	0.60027(15)	0.22230(12)	0.00915(9)	0.0189(4)
C23	0.55922(16)	0.07917(11)	0.15870(9)	0.0190(4)
C24	0.45733(17)	0.34563(12)	0.05184(10)	0.0230(4)
C25	0.65795(16)	0.25038(13)	0.14513(10)	0.0222(4)
C26	0.41484(16)	0.20365(12)	0.19988(10)	0.0218(4)
O31	0.43975(11)	0.16025(8)	0.42769(7)	0.0213(3)
O32	0.62835(12)	0.01799(9)	0.38801(7)	0.0218(3)
O33	0.68236(11)	0.03964(9)	0.55340(7)	0.0222(3)
O41	−0.12782(12)	0.47941(9)	0.11218(7)	0.0206(3)
O42	0.18117(12)	0.54117(9)	0.05373(7)	0.0230(3)
O43	0.06402(11)	0.33945(8)	0.07199(7)	0.0212(3)
O51	0.78655(11)	0.02770(15)	0.13515(9)	0.0391(4)
O52	−0.12323(13)	0.90227(12)	0.18228(8)	0.0404(4)
O53	0.12060(14)	0.95771(10)	0.12807(8)	0.0353(4)
O54	0.08004(13)	0.80894(10)	0.27389(7)	0.0326(3)
O61	0.54300(14)	0.69367(10)	0.22048(8)	0.0380(4)
O62	0.50302(15)	0.70856(11)	0.36466(9)	0.0372(4)
O63	0.66704(12)	0.57301(10)	0.35467(8)	0.0345(4)
O64	0.41132(13)	0.55340(11)	0.34101(9)	0.0358(4)
O1	0.19915(14)	0.59586(11)	0.19954(9)	0.0332(4)
O2	0.53803(14)	0.33541(10)	0.45173(9)	0.0310(4)
O3	0.04072(13)	0.16251(10)	0.05075(9)	0.0302(4)
O4	0.26383(13)	0.14861(10)	0.64970(8)	0.0310(3)
O5	0.67033(13)	0.10731(10)	0.69116(8)	0.0304(4)
O6	0.79220(14)	0.65718(10)	0.13810(9)	0.0338(4)
*O7	0.5372(9)	0.4560(6)	0.2059(6)	0.091(4)
*O8	−0.0086(13)	−0.0492(8)	0.7149(7)	0.104(5)

**Table S2.** Hydrogen bonds (Angstrom, Deg).

for: ia362 P -1 R = 0.03					
O1 -- H1A .. O51	0.846(18)	1.974(19)	2.799(2)	165.0(18)	.
O1 -- H1B .. O64	0.83(2)	2.08(2)	2.8709(18)	161(2)	.
O2 -- H2A .. O63	0.823(16)	2.49(2)	3.1444(19)	137(2)	.
O2 -- H2A .. O64	0.823(16)	2.164(19)	2.956(2)	161(3)	.
O2 -- H2B .. O62	0.831(13)	1.935(11)	2.7597(19)	172(2)	2_666
O3 -- H3A .. O53	0.841(18)	2.10(2)	2.8982(19)	159(2)	1_545
O3 -- H3B .. O51	0.839(12)	1.925(11)	2.7558(19)	171(2)	2_565
O5 -- H5A .. O62	0.832(19)	1.915(19)	2.745(2)	175.1(17)	2_666
O11 -- H11A .. O63	0.834(15)	1.888(14)	2.6977(15)	163.6(17)	1_455
O11 -- H11B .. O64	0.819(15)	1.915(16)	2.7129(16)	164.7(18)	2_566
O12 -- H12A .. O54	0.833(19)	1.84(2)	2.6695(17)	172.1(19)	2_566
O12 -- H12B .. O63	0.828(14)	1.979(14)	2.7409(15)	152.6(19)	2_666
O21 -- H21A .. O52	0.840(13)	1.860(15)	2.6773(16)	164.0(19)	2_565
O21 -- H21B .. O53	0.834(17)	1.968(17)	2.7585(16)	158.0(19)	1_545
O22 -- H22A .. O21	0.825(11)	2.46(3)	2.7813(16)	104(2)	2_655
O22 -- H22A .. O53	0.825(11)	2.205(14)	3.0154(18)	167(3)	2_665
O22 -- H22B .. O61	0.85(2)	1.79(2)	2.6280(18)	171(3)	2_665
O31 -- H31A .. N13	0.844(12)	2.006(13)	2.8416(16)	171(2)	.
O31 -- H31B .. O2	0.84(2)	1.84(2)	2.6745(18)	172.5(18)	.
O32 -- H32A .. N24	0.828(16)	2.056(14)	2.8718(16)	168.3(19)	.
O32 -- H32B .. O4	0.837(18)	1.861(18)	2.6952(19)	174(2)	2_656
O33 -- H33A .. N12	0.830(18)	2.062(19)	2.8798(17)	168(2)	1_655
O33 -- H33B .. O5	0.832(19)	1.856(19)	2.6827(17)	172.5(18)	.
O41 -- H41A .. O6	0.833(17)	1.822(17)	2.6537(19)	175.7(16)	1_455
O41 -- H41B .. N14	0.824(16)	2.074(15)	2.8815(16)	166.6(18)	.
O42 -- H42A .. O1	0.836(17)	1.868(18)	2.7013(18)	174.2(16)	.
O43 -- H43A .. N22	0.836(12)	2.062(14)	2.8851(16)	168(2)	.
O43 -- H43B .. O3	0.85(2)	1.86(2)	2.6960(18)	173(2)	.
C11 -- H11C .. O54	0.9900	2.5600	3.4872(17)	157.00	2_566
C11 -- H11D .. O2	0.9900	2.4900	3.4253(19)	158.00	1_455
C11 -- H11D .. O11	0.9900	2.4700	3.023(2)	115.00	.
C12 -- H12D .. O4	0.9900	2.4800	3.4275(19)	159.00	.
C13 -- H13A .. O63	0.9900	2.4300	3.3508(19)	155.00	1_455
C13 -- H13B .. O1	0.9900	2.4300	3.3914(19)	164.00	.
C14 -- H14A .. O8	0.9900	2.5700	3.456(11)	149.00	2_556
C21 -- H21C .. O6	0.9900	2.4500	3.403(2)	161.00	2_665
C21 -- H21D .. O53	0.9900	2.5100	3.429(2)	155.00	1_545
C22 -- H22C .. O3	0.9900	2.4800	3.4004(18)	154.00	1_655
C23 -- H23A .. O5	0.9900	2.4300	3.3984(19)	166.00	2_656
C23 -- H23A .. O22	0.9900	2.4800	3.042(2)	115.00	2_655
C24 -- H24A .. O7	0.9900	2.5200	3.412(9)	149.00	.
C25 -- H25B .. O7	0.9900	2.5300	3.419(9)	149.00	.

## Figures and Tables

**Table 1:** Nitrogen adsorption on nickel(II) MOF-type supramolecule.

P/P_O_	1/[W(P/P_O_)-1)]	Vol.@STP	P/P_O_/W [g/g]
5.80E-02	1.72E+00	28.6635	1.62E+00
1.18E-01	2.24E+00	44.7618	2.11E+00
1.81E-01	2.87E+00	61.7584	2.35E+00
2.43E-01	3.27E+00	78.4228	2.47E+00
3.05E-01	3.66E+00	95.9876	2.54E+00

**Table 2:** Crystal data.

Formula	C_12_ H_52_ N_8_ Ni_2_ O_24.55_ S_2_
Formula weight	882.92
Crystal system	triclinic
Space group	P-1(No.2)
a, b, c [Angstrom]	9.2053(4) 13.2964(5)15.8998(6)
alpha, beta, gamma [deg]	66.296(2) 89.400(2) 89.441(2)
V [Ang**3]	1781.78(13)
Z	2
D(calc) [g/cm^−3^]	1.6457(1)
Mu(MoKa) [ /mm ]	1.272
F(000)	929
Crystal Size [mm]	0.34 × 0.42 × 0.43
Data collection	
Temperature (K)	200
Radiation [Angstrom]	MoKa 0.71073
Theta Min–Max [Deg]	1.4, 28.4
Dataset	−12: 12; −17: 17; −21: 21
Tot., Uniq. Data, R(int)	79742, 8877, 0.022
Observed Data [I > 2.0 sigma(I)]	7704
Refinement	
Nref, Npar	8877, 571
R, wR2, S	0.0258, 0.0806, 1.03
